# Identification and Validation of an Explainable Prediction Model of Sepsis in Patients With Intracerebral Hemorrhage: Multicenter Retrospective Study

**DOI:** 10.2196/71413

**Published:** 2025-04-28

**Authors:** Xianglin Liu, Zhihua Huang, Yizhi Guo, Yandeng Li, Jianming Zhu, Jun Wen, Yunchun Gao, Jianyi Liu

**Affiliations:** 1 Changde Hospital, Xiangya School of Medicine Central South University (The First People's Hospital of Changde City) Changde China

**Keywords:** intracerebral hemorrhage, machine learning, sepsis, prediction model, SHAP, Shapley Additive Explanations

## Abstract

**Background:**

Sepsis is a life-threatening condition frequently observed in patients with intracerebral hemorrhage (ICH) who are critically ill. Early and accurate identification and prediction of sepsis are crucial. Machine learning (ML)–based predictive models exhibit promising sepsis prediction capabilities in emergency settings. However, their application in predicting sepsis among patients with ICH is still limited.

**Objective:**

The aim of the study is to develop an ML-driven risk calculator for early prediction of sepsis in patients with ICH who are critically ill and to clarify feature importance and explain the model using the Shapley Additive Explanations method.

**Methods:**

Patients with ICH admitted to the intensive care unit (ICU) from the Medical Information Mart for Intensive Care IV database between 2008 and 2022 were divided into training and internal test sets. The external test was performed using the eICU Collaborative Research Database, which includes over 200,000 ICU admissions across the United States between 2014 and 2015. Sepsis following ICU admission was identified using Sepsis-3.0 through clinical diagnosis combining elevation of the Sequential Organ Failure Assessment by ≥2 points with suspected infection. The Boruta algorithm was used for feature selection, confirming 29 features. Nine ML algorithms were used to construct the prediction models. Predictive performance was compared using several evaluation metrics, including the area under the receiver operating characteristic curve (AUC). The Shapley Additive Explanations technique was used to interpret the final model, and a web-based risk calculator was constructed for clinical practice.

**Results:**

Overall, 2414 patients with ICH were enrolled from the Medical Information Mart for Intensive Care IV database, with 1689 and 725 patients assigned to the training and internal test sets, respectively. An external test set of 2806 patients with ICH from the eICU database was used. Among the 9 ML models tested, the categorical boosting (CatBoost) model demonstrated the best discriminative ability. After reducing features based on their importance, an explainable final CatBoost model was developed using 8 features. The final model accurately predicted sepsis in internal (AUC=0.812) and external (AUC=0.771) tests.

**Conclusions:**

We constructed a web-based risk calculator with 8 features based on the CatBoost model to assist clinicians in identifying people at high risk for sepsis in patients with ICH who are critically ill.

## Introduction

Patients with intracerebral hemorrhage (ICH) who are critically ill, namely, those with life-threatening conditions that require intensive medical intervention and continuous monitoring in the intensive care unit (ICU), are highly susceptible to various clinical complications. Posthemorrhagic infection is the most prevalent complication, with an incidence as high as 58% [1,2]. Sepsis is a life-threatening condition characterized by acute organ dysfunction caused by a dysregulated host response to infection [3]. Most patients with ICH who develop infections deteriorate rapidly and progress to sepsis due to systemic metabolic disorders, stress caused by excessive inflammatory factor release, and immunosuppression [4,5]. Consequently, patients with ICH exhibit a higher incidence of sepsis than other ICU populations, with approximately 28% of patients with ICH developing secondary sepsis [6]. Moreover, sepsis is associated with significantly worse prognoses, leading to a 2-fold increase in mortality rates during hospitalization (36.7% vs 18.8%) and at 3 months after admission (56.5% vs 28.5%) [4,7]. A prospective cohort study reported that the early detection of sepsis promoted early treatment, reducing in-hospital mortality from sepsis by 23% [8]. Therefore, early and accurate identification of patients at high risk for sepsis is essential for initiating timely therapeutic interventions and improving clinical outcomes.

However, the pathophysiological changes induced by ICH itself present unique challenges for early sepsis recognition [7,9]. First, primary disease manifestations such as impaired consciousness, tachypnea, blood pressure fluctuations, and thermoregulatory abnormalities (fever or hypothermia) substantially overlap with typical sepsis symptoms (high fever, tachycardia, and mental lethargy). Second, stress responses triggered by ICH may cause leukocytosis and abnormal inflammatory markers, such as elevated C-reactive protein and procalcitonin, which closely resemble diagnostic criteria of sepsis (C-reactive protein>50 mg/L and procalcitonin>0.5 µg/L). Additionally, impaired consciousness often hinders accurate symptom reporting. These overlapping features and diagnostic ambiguities render the early identification of sepsis secondary to cerebral hemorrhage a significant clinical challenge.

Artificial intelligence, a branch of computer science, focuses on developing systems capable of cognitive abilities that surpass human capacities in perception, learning, problem-solving, and decision-making [10]. Machine learning (ML), a subset of artificial intelligence, enables computers to learn from data and improve performance without explicit programming. When provided with sufficient high-quality data, ML algorithms can effectively learn to make predictions or solve complex problems. Consequently, ML-based predictive models using electronic medical records (EMRs) have gained significant clinical attention owing to their potential to enhance diagnostic accuracy, expedite decision-making, and refine prognosis estimation, as demonstrated in various acute conditions such as acute kidney injury [11], atrial fibrillation [12], and heart failure [13]. Although existing ML models exhibit promising sepsis prediction capabilities in general ICU and emergency settings [14], critical gaps remain unaddressed. First, no ML tools have been specifically designed for sepsis detection in ICH populations. Second, most models rely on single-center datasets, which lack external validation [15], raising concerns about their generalizability across institutions with varying patient demographics, clinical protocols, and data infrastructures. Model performance often deteriorates when applied to external cohorts due to dataset heterogeneity. Critically, despite the demonstrated potential of ML models in sepsis prediction, their inherent complexity, which often renders them “black boxes” [16], coupled with the absence of successful real-world clinical integration, continues to hinder their practical adoption.

Thus, to address these limitations, we conducted a multicenter retrospective study using 2 distinct critical care databases: the Medical Information Mart for Intensive Care IV (MIMIC-IV) database for model development and the eICU Collaborative Research Database for external validation. Our objectives were twofold: (1) to develop an ML-driven web-based risk calculator for early prediction of sepsis in patients with ICH who are critically ill and (2) to clarify feature importance and explain the model using the Shapley Additive Explanations (SHAP) method [17], a technique for interpreting ML models and visualizing individual variable predictions. This dual-phase approach aims to bridge the translational gap between computational research and clinical application in neurocritical care.

## Methods

### Ethical Considerations

The MIMIC-IV and eICU databases were deidentified, anonymized, and approved for sharing by the institutional review boards of both Beth Israel Deaconess Medical Center and the Massachusetts Institute of Technology. Data access was granted to an investigator after the completion of a National Institutes of Health course and successful passing of the associated human research participant protection examination. Given that the data are accessible to the public through the MIMIC-IV and eICU database, the need for ethical approval and informed consent was waived. The contributing author, JL, obtained the necessary authorization to access the anonymized dataset (ID: 60367406) and oversaw the meticulous data extraction process.

### Data Source

The prediction model was developed by deploying data from patients who were diagnosed with ICH and admitted to the ICU, sourced from the MIMIC-IV (version 3.1). This anonymized and publicly accessible database includes health records of patients admitted to the critical care units at Beth Israel Deaconess Medical Center between 2008 and 2022 [18]. Additionally, external testing was performed using the eICU Collaborative Research Database, a multicenter database that includes deidentified health data from over 200,000 ICU admissions across 208 hospitals in the United States between 2014 and 2015 [19].

### Data Collection and Processing

Patients who met the following criteria were included in this study: (1) a diagnosis of ICH based on the *ICD-9* (*International Classification of Diseases, Ninth Revision*) and *ICD-10* (*International Statistical Classification of Diseases, Tenth Revision*; *ICD-9*: 431 and *ICD-10*: I610-I619 and I629); (2) age >18 years; (3) ICU admission exceeding 1 day; and (4) for patients with multiple ICU admissions, only the first ICH-related admission was considered. Figure S1 in Multimedia Appendix 1 outlines the patient screening process.

Data for patients with ICH admitted to the ICU within the first 24 hours were extracted using Structured Query Language from 2 databases. The extracted variables included the following: (1) demographic details such as age, sex, race, and weight; (2) comorbidities, including myocardial infarction, heart failure, diabetes mellitus, hypertension, and malignant neoplasms; (3) laboratory parameters, including mean corpuscular volume, partial thromboplastin time, magnesium, phosphate, international normalized ratio, prothrombin time, red blood cell distribution width, blood urea nitrogen, red blood cells, hemoglobin, hematocrit, white blood cells (WBCs), platelets, creatinine, glucose, anion gap, potassium, sodium, calcium, and chloride; (4) vital signs, including heart rate, respiratory rate, systolic blood pressure, diastolic blood pressure, mean arterial pressure, temperature, and percutaneous arterial oxygen saturation (SpO_2_); (5) interventions, such as mechanical ventilation (MV) and continuous renal replacement therapy; and (6) clinical severity indices, including the Glasgow Coma Scale (GCS), Sequential Organ Failure Assessment (SOFA), and Simplified Acute Physiology Score II (SAPSII). The average value from the first day was used for variables measured multiple times. To minimize the effect of missing data on model development, variables with >20% missing values were excluded, while those with <20% missing values were imputed using the *MICE* package in R [20]. The primary outcome was sepsis that manifested subsequent to ICU admission. Its clinical diagnosis was established in accordance with Sepsis 3.0 criteria [3], entailing an elevation of the SOFA score by ≥2 points in conjunction with a suspected infection. Suspected infection required temporal alignment between bacterial culture collection and antibiotic administration: (1) microbial sampling (blood or tissue cultures) within 24 hours preceding antibiotic initiation or (2) antibiotic commencement within 72 hours following initial microbial sampling.

### Feature Selection

After excluding features with >20% missing values, 41 potential candidate predictors were considered for model development. To optimize performance, reduce dimensionality, and improve interpretability, the Boruta algorithm was used for feature selection. This technique, based on the random forest (RF) algorithm, identifies relevant features by comparing the importance of actual variables (their contribution to the predictive accuracy of the model) with the importance achieved randomly using permuted copies of the attributes. Features identified through this selection process were used for model development and to determine the most important and informative predictors. To address collinearity, which complicates the evaluation of the unique contribution of each feature to the outcome, the collinearity of the identified features was assessed by deploying a pairwise Spearman correlation matrix and applying a threshold of *r*>0.8 (Figure S2 in Multimedia Appendix 1).

### Model Development and Comparison

Data from the MIMIC-IV database were divided by 70% and 30% for the training and internal tests, respectively, to reduce the risk of overfitting. Data from the eICU database served as the external test set. Nine ML models were used to predict sepsis in patients with ICH who are critically ill: decision tree, logistic regression, extreme gradient boosting, categorical boosting (CatBoost), RF, light gradient boosting machine (LightGBM), support vector machine, k-nearest neighbor, and adaptive boosting. The prediction model was optimized through a combination of grid search, manual fine-tuning, and 10-fold cross-validations in the training set. Model performance was assessed using various metrics such as the area under the receiver operating characteristic curve (AUC), sensitivity, specificity, precision, accuracy, false positive rate, false negative rate, and *F*_1_-score. Additional evaluations included decision curve analysis and precision-recall curve analysis.

### Model Explanation

To ensure an accurate interpretation of the ML model, the SHAP technique was applied to overcome the “black-box” challenge. The SHAP method provides both global and local explanations for the model. The global explanation provides consistent and accurate attribution values for each feature, highlighting the correlations among input features and sepsis. Conversely, the local explanation illustrates a specific prediction for an individual patient by using their specific data.

### Clinical Application

The final model was deployed as a user-friendly web application to enhance clinical applicability. Upon inputting relevant patient data, it instantly calculates the probability of sepsis and generates individualized Shapley force plots for outcome interpretation.

### Statistical Analysis

Data analyses were performed using R (version 4.4.1; R Foundation for Statistical Computing) and the DecisionLinnc1.0 (DecisionLinnc Core Team) software. DecisionLinnc1.0 is a versatile platform that integrates various programming language environments, enabling data processing, analysis, and ML through an intuitive visual interface. Continuous variables were expressed as mean (SD) or median (IQR), while categorical variables were presented as counts (n) and percentages (%). For group comparisons, the Mann-Whitney *U* test or Student *t* test was used for continuous variables, and the Fisher exact test or the chi-square test for categorical variables. A 2-tailed *P* value of <.05 was considered significant.

## Results

### Patient Characteristics

Overall, 2414 patients with ICH were enrolled from the MIMIC-IV database, with 1689 patients designated to the training set and 725 to the internal test set. Based on whether sepsis occurred after ICU admission, patients were classified into sepsis (n=974 patients) and nonsepsis (n=1440 patients) groups. Table 1 illustrates the demographic and clinical characteristics that differed between these 2 groups. Specifically, compared to the nonsepsis group, the sepsis group exhibited higher SOFA and SAPSII scores, along with increased rates of MV and continuous renal replacement therapy. Additionally, the sepsis group exhibited elevated levels of WBC, heart rate, SpO_2_, blood urea nitrogen, and glucose than the nonsepsis group. Furthermore, an external test set including 2806 patients with ICH from the eICU database was used. Table S1 in Multimedia Appendix 1 shows a comparison of the demographic and clinical variables across the training, internal test, and external test sets.

**Table 1:** Comparison of demographic and clinical characteristics between nonsepsis and sepsis.

Variables				Overall (N=2414)		Nonsepsis (n=1440)		Sepsis (n=974)		*P* value	
**Demographic**											
	Age (years), median (IQR)			70.0 (58.0-81.0)		71.0 (59.0-82.0)		68.0 (57.0-79.0)		<.001	
	Weight (kg), median (IQR)			76.8 (64.3-90.7)		76.0 (64.0-90.1)		78.0 (65.0-91.7)		.07	
	Male, n (%)			1299 (53.8)		757 (52.6)		542 (55.6)		.14	
	**Race, n (%)**									<.001	
		Black	216 (8.9)		136 (9.4)		80 (8.2)				
		White	1388 (57.5)		876 (60.8)		512 (52.6)				
		Other	810 (33.6)		428 (29.7)		382 (39.2)				
**Clinical severity, median (IQR)**											
	SOFA^a^			3.0 (2.0-5.0)		2.0 (1.0-4.0)		4.0 (2.0-6.0)		<.001	
	SAPSII^b^			32.0 (25.0-40.0)		30.0 (24.0-38.0)		35.0 (28.0-44.0)		<.001	
	GCS^c^			14.0 (11.0-15.0)		14.0 (12.0-15.0)		14.0 (10.0-15.0)		.02	
**Vital signs, median (IQR)**											<.001
	HR^d^ (beats per minute)			79.7 (70.6-89.1)		78.0 (69.2-87.4)		82.2 (72.9-91.9)			
	DBP^e^ (mm Hg)			70.2 (62.7-78.6)		71.0 (63.5-78.9)		68.9 (61.3-77.8)			
	SBP^f^ (mm Hg)			129.6 (119.2-138.8)		130.5 (121.3-138.9)		127.9 (116.0-138.5)			
	MAP^g^ (mm Hg)			85.5 (77.9-93.1)		86.5 (78.9-93.8)		83.4 (76.0-92.0)			
	RR^h^ (breath per minute)			18.3 (16.5-20.5)		18.0 (16.2-20.0)		18.8 (17.0-21.3)			
	SpO_2_^i^ (%)			97.1 (95.9-98.6)		96.8 (95.6-98.1)		97.8 (96.2-99.1)			
	Temperature (°C)			36.9 (36.7-37.3)		36.9 (36.7-37.1)		37.1 (36.8-37.5)			
**Laboratory parameters, median (IQR)**											
	Hematocrit (%)			36.9 (33.0-40.3)		37.5 (33.9-40.6)		35.9 (31.9-39.8)		<.001	
	Hemoglobin (g/dL)			12.3 (10.9-13.5)		12.5 (11.2-13.6)		11.9 (10.4-13.2)		<.001	
	Platelet (10^9^/L)			206.8 (162.0-258.0)		211.0 (167.0-258.0)		200.8 (152.0-258.0)		.004	
	RDW^j^ (%)			13.8 (13.1-14.7)		13.6 (13.0-14.5)		14.0 (13.2-15.0)		<.001	
	RBC^k^ (10^9^/L)			4.1 (3.6-4.5)		4.1 (3.7-4.5)		4.0 (3.5-4.4)		<.001	
	WBC^l^ (10^9^/L)			10.5 (8.0-13.3)		9.7 (7.7-12.2)		11.6 (9.1-14.8)		<.001	
	MCV^m^ (fl)			91.0 (87.0-94.5)		91.0 (87.5-94.0)		91.0 (87.0-95.0)		.89	
	Anion gap (mmol/L)			14.0 (12.0-16.0)		14.0 (12.0-16.0)		14.0 (12.3-16.0)		.10	
	Calcium (mg/dL)			8.8 (8.4-9.1)		8.9 (8.5-9.2)		8.6 (8.2-9.0)		<.001	
	Chloride (mmol/L)			104.0 (101.5-107.0)		104.0 (101.0-106.0)		105.0 (102.0-108.7)		<.001	
	Glucose (mg/dL)			130.0 (109.0-157.0)		125.0 (106.0-150.0)		136.5 (115.5-167.0)		<.001	
	Potassium (mmol/L)			3.9 (3.7-4.2)		3.9 (3.7-4.2)		3.9 (3.6-4.3)		.71	
	Sodium (mmol/L)			140.0 (137.5-142.0)		139.5 (137.0-142.0)		140.0 (137.5-143.0)		<.001	
	Magnesium (mg/dL)			2.0 (1.8-2.1)		2.0 (1.8-2.1)		2.0 (1.8-2.1)		.93	
	Phosphate (mg/dL)			3.2 (2.7-3.7)		3.2 (2.8-3.7)		3.2 (2.7-3.8)		.51	
	INRPT^n^			1.2 (1.1-1.3)		1.1 (1.1-1.2)		1.2 (1.1-1.3)		<.001	
	PT^o^ (seconds)			12.6 (11.7-13.9)		12.4 (11.6-13.7)		12.8 (11.9-14.3)		<.001	
	PTT^p^ (seconds)			28.1 (25.7-31.2)		28.0 (25.8-31.2)		28.4 (25.5-31.2)		.21	
	Creatinine (mg/dL)			0.9 (0.7-1.1)		0.9 (0.7-1.1)		0.9 (0.7-1.2)		<.001	
	BUN^q^ (mg/dL)			16.0 (12.0-22.0)		15.5 (12.0-21.0)		16.7 (12.3-24.0)		<.001	
**Comorbidities, n (%)**											
	HTN^r^			1513 (62.7)		938 (65.1)		575 (59)		.002	
	Cancer			364 (15.1)		241 (16.7)		123 (12.6)		.006	
	Diabetes			600 (24.9)		351 (24.4)		249 (25.6)		.51	
	HF^s^			297 (12.3)		142 (9.9)		155 (15.9)		<.001	
	MI^t^			87 (3.6)		27 (1.9)		60 (6.2)		<.001	
**Interventions, n (%)**											<.001
	CRRT^u^			2378 (98.5)		1440 (100)		938 (96.3)			
	MV^v^			1222 (50.6)		804 (55.8)		418 (42.9)			

^a^SOFA: Sequential Organ Failure Assessment.

^b^SAPSII: Simplified Acute Physiology Score II.

^c^GCS: Glasgow Coma Scale.

^d^HR: heart rate.

^e^DBP: diastolic blood pressure.

^f^SBP: systolic blood pressure.

^g^MAP: mean arterial pressure.

^h^RR: respiratory rate.

^i^SpO_2_: percutaneous arterial oxygen saturation.

^j^RDW: red blood cell distribution width.

^k^RBC: red blood cell.

^l^WBC: white blood cell.

^m^MCV: mean corpuscular volume.

^n^INRPT: international normalized ratio.

^o^PT: prothrombin time.

^p^PTT: partial thromboplastin time.

^q^BUN: blood urea nitrogen.

^r^HTN: hypertension.

^s^HF: heart failure.

^t^MI: myocardial infarction.

^u^CRRT: continuous renal replacement therapy.

^v^MV: mechanical ventilation.

### Model Development and Performance Comparison

**Figure 1:**
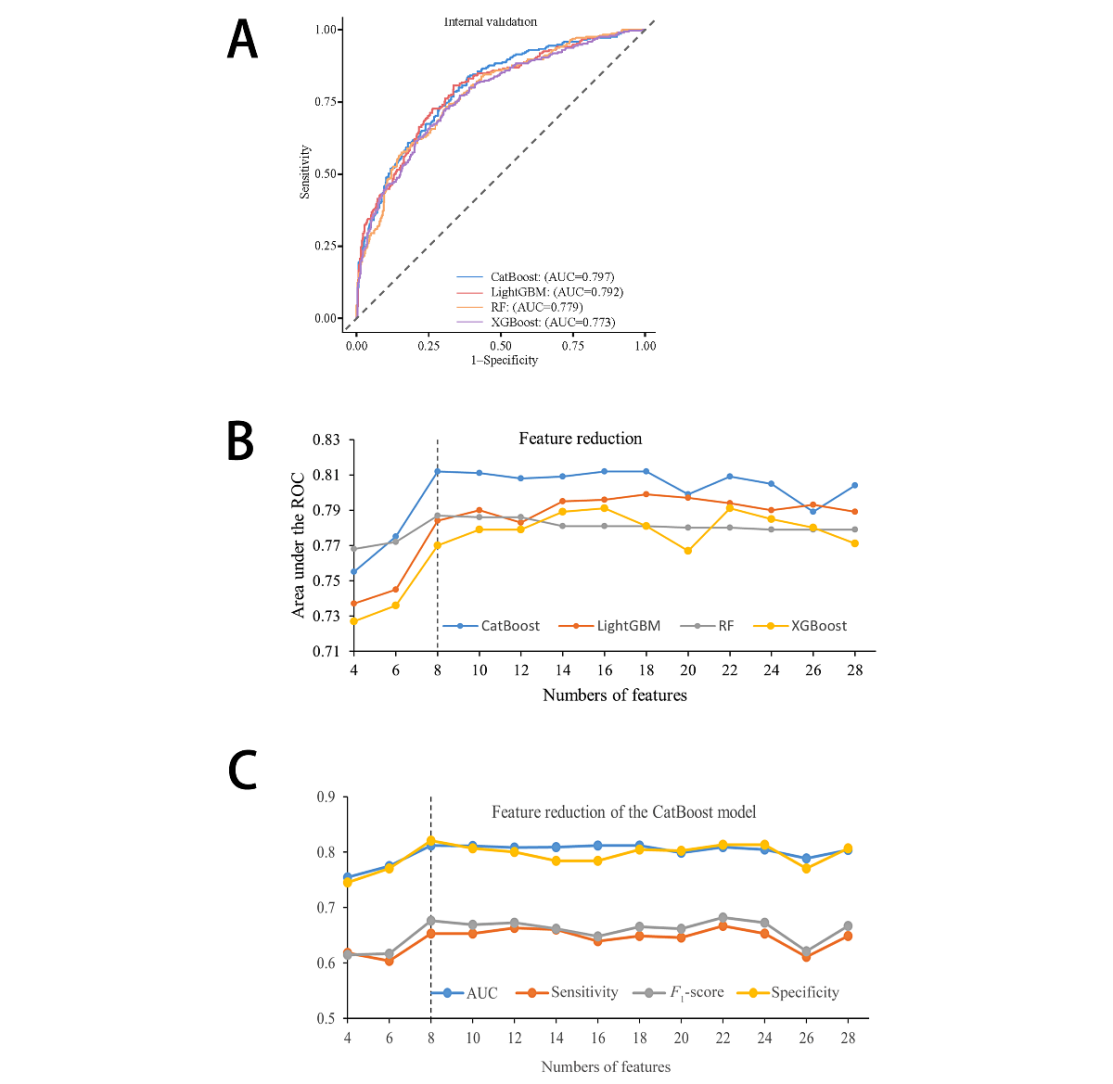
Performance of ML models predicting sepsis. (A) ROC curves for the top 4 best-performing ML models. (B) AUCs of the top 4 best-performing ML models with varied numbers of features. (C) AUC, sensitivity, specificity, and F1-score for the CatBoost model with varying numbers of features. AUC: area under the receiver operating characteristic curve; CatBoost: categorical boosting; LightGBM: light gradient boosting machine; ML: machine learning; RF: random forest; ROC: receiver operating characteristic; XGBoost: extreme gradient boosting.

The Boruta algorithm was used to identify relevant features, with their importance displayed in Figure S3 in Multimedia Appendix 1. Based on the 29 features identified via Boruta, 9 ML models were developed to predict sepsis in patients with ICH following ICU admission. Among these models, the CatBoost model exhibited the best predictive performance for sepsis, followed closely by the LightGBM and RF models. Table S2 in Multimedia Appendix 1 shows the performance of the 9 models. Additionally, Figure 1A presents the receiver operating characteristic curves for the top 4 performing models. Figure 1B shows that by reducing features according to their importance ranking, the changes in AUCs for these 4 top models demonstrated that the CatBoost model consistently maintained nearly optimal predictive ability. Therefore, the CatBoost model outperformed the other models in sepsis prediction. Figure 1C and Table S3 in Multimedia Appendix 1 show the performance of the CatBoost model with varying numbers of features. Sensitivity, specificity, accuracy, and *F*_1_-score are calculated at the optimal cutoff value that maximizes the Youden index.

### Identification of the Final Model

**Figure 2:**
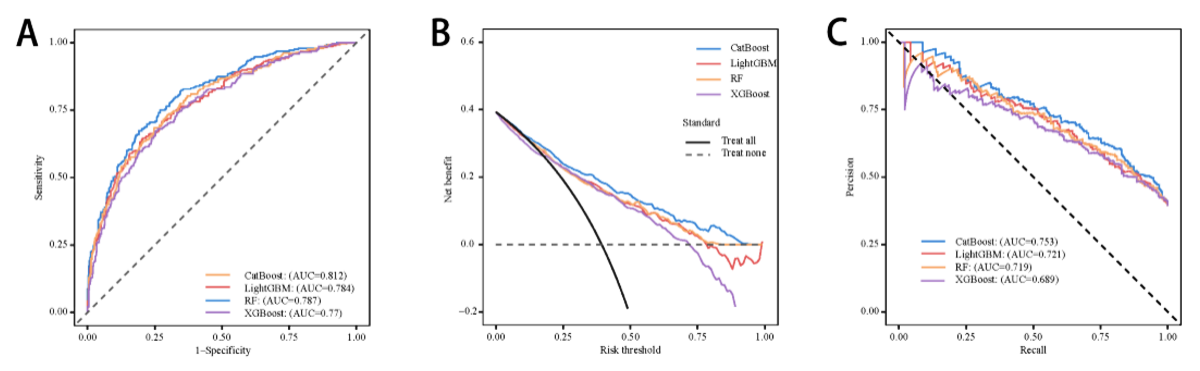
Predictive performance of the top 4 best-performing machine learning models with 8 features. (A) Receiver operating characteristic, (B) decision curve analysis, and (C) precision-recall curves. AUC: area under the receiver operating characteristic curve; CatBoost: categorical boosting; LightGBM: light gradient boosting machine; RF: random forest; XGBoost: extreme gradient boosting.

During the feature reduction process for the CatBoost model, the final model was selected. Figure 1 and Table S3 in Multimedia Appendix 1 illustrate the top 8 features—SOFA, SAPSII, calcium, chloride, WBC, SpO_2_, temperature, and MV—provided optimal results with minimal features necessary for predicting sepsis in ICU-admitted patients with ICH. Therefore, the 8-feature CatBoost model was selected as the final model for further analysis. The finalization of the hyperparameters for the CatBoost model as learning_rate=0.1, depth=10, and iterations=100; LightGBM as learning_rate=0.1; and RF as mtry=100 and depth=3. This final model achieved an AUC of 0.812 (95% CI 0.780-0.844), with a sensitivity, specificity, accuracy, and *F*_1_-score of 0.653, 0.820, 0.754, and 0.676, respectively, for sepsis prediction in patients with ICH who are critically ill. Figure 2 shows the receiver operating characteristic, decision curve analysis, and precision-recall curves of the final model. These results further validate the reliability and accuracy of the final CatBoost model in predicting sepsis.

The predictive values of SOFA and SAPSII, which all reflect the severity of the condition of the patients, were further examined before being compared with that of the 8-feature final model. Figure S4A in Multimedia Appendix 1 illustrates that SOFA and SAPSII performed worse in the internal test than that in the final model, respectively. Figure S4B in Multimedia Appendix 1 also indicates that the final model offered enhanced clinical utility compared to that of the SOFA and SAPSII scores, respectively.

### External Test of the Final Model

For the external test, the final model achieved an AUC of 0.771 (95% CI 0.752-0.790), comparable to that observed during the internal test, indicating that the final model consistently maintained high predictive performance across both test datasets. Additionally, we performed additional validation of the final model specifically in the subgroup of patients with a GCS score of ≤8. The results demonstrated robust predictive performance, with AUCs of 0.808 (95% CI 0.726-0.890) in the internal test set and 0.764 (95% CI 0.718-0.809) in the external test set. These findings indicate that our model maintains stable predictive accuracy even in patients with severe ICH, and it addresses the clinical need for sepsis prediction in heterogeneous ICH populations.

### Model Explanation

**Figure 3:**
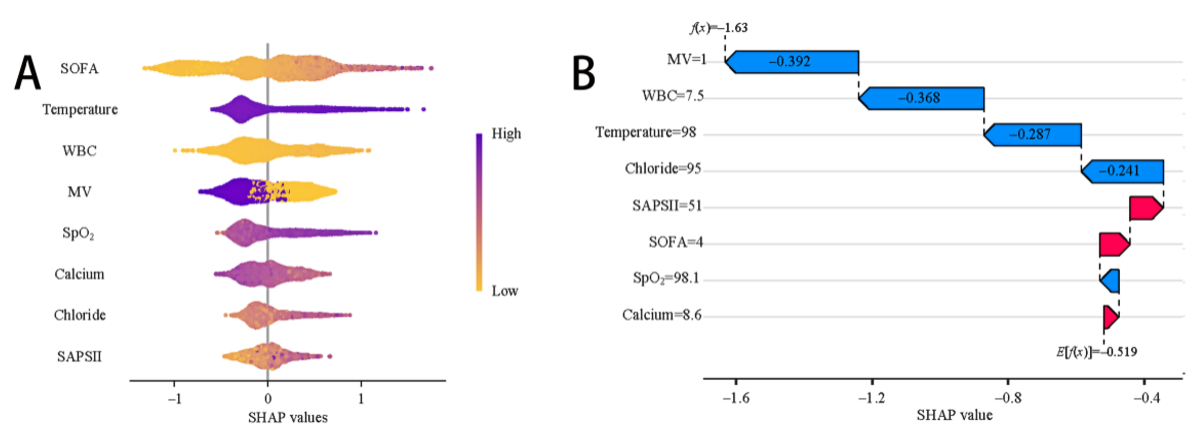
Model explanation using the SHAP method. (A) SHAP summary dot plot and (B) waterfall plot. MV: mechanical ventilation; SAPSII: Simplified Acute Physiology Score II; SHAP: Shapley Additive Explanations; SOFA: Sequential Organ Failure Assessment; SpO_2_: percutaneous arterial oxygen saturation; WBC: white blood cell.

Figure 3A illustrates a detailed swarm plot displaying the variables used in the CatBoost model. The horizontal axis represents SHAP values, whereas the vertical axis ranks features based on their cumulative effect on the SHAP value. Each data point represents a specific instance, with its position on the x-axis reflecting the SHAP value for that particular instance and feature. Figure 3B highlights a comprehensive case study illustrating the prediction process of the model for a specific patient. In this visualization, red markers indicate positive contributions to the prediction, whereas blue markers represent negative influences. The *f*(*x*) value corresponds to the SHAP value of each factor. For this patient, the CatBoost model predicted a lower risk of sepsis compared with that of the baseline.

### Convenient Application for Clinical Utility

The final prediction model was incorporated into the web application to improve its applicability in clinical settings [21]. By inputting the actual values of the 8 required features, the application generates an automated prediction of sepsis risk for individual patients with ICH. Furthermore, a force plot is presented for each patient, visually demonstrating the features that influence the sepsis prediction. Specifically, blue features on the right indicate those pushing the prediction toward “nonsepsis,” whereas red features on the left indicate those pushing it toward “sepsis.”

## Discussion

### Principal Findings

The aims of this study were to assess the risk of sepsis developing in patients with ICH and to use ML methods to provide an in-depth explanation of the model’s decision-making and its impact on features. This multicenter, retrospective analysis, to our knowledge, represents the first study to investigate and compare 9 ML models for predicting sepsis among patients with ICH who are critically ill. By integrating ML algorithms alongside clinical and laboratory data obtained from EMR, we identified key predictive risk factors and developed a targeted sepsis prediction model specifically for patients with ICH admitted to the ICU. Finally, we translated the best models into a web-based risk calculator to help clinicians identify patients at high risk.

### Comparison to Prior Work

Accurately identifying sepsis and predicting patients at risk of developing it are clinically crucial for improving treatment outcomes [22-24]. Current approaches to identifying patients with sepsis have focused on biomarkers and automated clinical decision rules, including the SOFA criteria [25,26]. However, implementing a single analyte in clinical practice remains challenging owing to the heterogeneity underlying the pathophysiology of sepsis [3,4,27]. Additionally, concerns have emerged about the poor sensitivity of the SOFA score, which may result in delays in sepsis detection [28]. Furthermore, most of the biomarkers examined lacked discriminative power or clinical relevance [29,30]. The ML technique, a robust computational tool capable of managing complex and large datasets, offers a novel approach to early sepsis identification, demonstrating excellent individual performance. It can manage highly variable datasets and understand the intricate correlation among variables in a flexible and adaptable manner. By integrating easily accessible EMR data, which enhances accuracy for clinicians and researchers, with sophisticated ML algorithms, the development of clinical prediction models can be improved [31]. Among the 9 ML models evaluated in this study, the CatBoost model achieved the highest AUC value, demonstrating strong net benefit and optimal threshold probability during feature reduction. As a decision tree–based algorithm, CatBoost is well-suited for ML tasks involving categorical and heterogeneous data [32]. Numerous studies have confirmed the strong predictive value of the CatBoost in the medical field [33-35]. In this study, the CatBoost algorithm was used to develop a final model incorporating 8 features. These features, which can be easily obtained or assessed within the first 24 hours of ICU admission, make the model a promising tool for early sepsis detection in patients with ICH during their ICU stay, even for those who have not yet met the criteria for sepsis at the time of data collection. In clinical practice, sepsis poses a particularly significant threat to patients with severe ICH, exacerbating therapeutic challenges—especially in those presenting with a GCS score ≤8 and requiring endotracheal intubation. Consequently, early identification of individuals at risk of sepsis within this population with critical illness holds substantial clinical importance. In this study, we conducted subgroup analyses focusing on patients with ICH with a GCS score ≤8 and observed that the final predictive model retained consistent accuracy in predicting sepsis risk among severe ICH cases. These findings demonstrate that our model effectively addresses the clinical need for sepsis prediction in heterogeneous ICH populations, underscoring its robust applicability in clinical settings.

Given the lack of established guidelines or consensus on feature selection for prediction models, the optimal number of features to include remains unclear. While incorporating more features may offer additional information to the model, excessive inclusion can hinder its clinical applicability, and noncausal features may reduce prediction accuracy [36]. To address this challenge, the Boruta method was used for feature selection. Our final model, designed to be a simple and convenient ML prediction tool, could easily be used to facilitate clinical decision-making in patients with ICH who are critically ill. Compared with traditional single markers, the final model identified in this study demonstrates superior predictive ability for sepsis. Given the crucial role of SOFA and SAPSII in diagnosing sepsis, both are objective clinical criteria used to assess the severity of the condition of the patient. Including these 2 features is beneficial for enhancing the predictive ability of the final model [37,38]. When comparing the predictive power of the final model to that of the SOFA and SAPSII, we observed that the final model outperformed each traditional marker. Several predictive covariates in the final model, including temperature, are clinically recognized for their relevance in sepsis detection. As expected, SOFA correlated with model performance, owing to its frequent inclusion in sepsis definitions. However, other factors, such as calcium, which are not typically part of these criteria, also show strong univariate predictors and are included in various ML models for sepsis prediction [14]. Disease severity is correlated with decreased serum total and ionic calcium levels, with hypocalcemia worsening as infection severity increases [39]. This highlights the importance of blood calcium levels in predicting sepsis risk among patients with critical illness. Laboratory values are often overlooked in early warning scores [3], but our findings suggest that these scores may miss crucial predictive information. Although the mechanism connecting chloride to sepsis remains unclear, previous studies report a correlation between chloride and sepsis [40]. Additionally, MV increases the risk of sepsis [41]. Therefore, these clinical variables could enhance the final model, and their combined application may offer superior sepsis prediction compared to that of a single marker.

Our final model demonstrated robust performance in internal and external tests, achieving AUCs of 0.812 and 0.771, respectively. Previous studies have explored ML models for sepsis prediction. The Epic Sepsis Model, a widely used early warning system in the United States [42], achieved an AUC of 0.63 in an external test with a cohort of 27,697 individuals, indicating poor discrimination and calibration [43]. In contrast, Wang et al [44] developed a prediction model using supervised learning on 4449 ICU patients with infections, achieving an AUC of 0.91 for sepsis prediction. Rafiei et al [45] also used a convolutional neural network to predict sepsis, incorporating onset time and achieving an AUC >0.8. However, existing studies primarily focused on sepsis in the general population. Due to the significant variability in the causes and progression of sepsis, along with the heightened risk of sepsis in patients with ICH, establishing prediction models specifically for ICU patients with ICH is crucial. Our final model, obtained through a comparison of 9 ML models and feature reduction, accurately predicted sepsis in patients with ICH who are critically ill throughout their ICU stay. The ML technique is often considered a “black box” owing to the lack of transparency in the prediction process. This opacity may cause clinicians to hesitate in relying on ML-based medical decisions. To enhance understanding and clinical applicability, the SHAP method was used to clarify the predictions of the model. The SHAP technique offers a global explanation of the overall functionality of the model and a local explanation that reveals how individual predictions are made on the basis of specific patient data. Furthermore, we provided a web-based risk calculator to help clinicians identify patients with high risk for sepsis, further underscoring its potential for clinical applicability and its benefits.

### Strengths and Limitations

We acknowledge some limitations in this study. First, our sepsis prediction model was developed in an ICU setting without considering the diverse etiologies of sepsis. Due to the complexity of sepsis pathophysiological mechanisms, whether the model can reliably predict various sepsis types remains unclear. Second, the model was constructed using data from American populations, leaving its generalizability to global populations uncertain. However, with the use of a multicenter eICU database for external test, our findings may suggest potential generalizability, though further assessment is needed to confirm this. Third, our final model only predicts sepsis occurrence, not its timing. Therefore, further research is needed to investigate the prediction of sepsis timing, particularly within a 24- or 48-hour window before its occurrence. Fourth, the absence of pathogen culture results introduces uncertainty in identifying the source of infection and the specific pathogens involved. This limitation could potentially compromise the precise determination of sepsis etiology and introduce biases in the practical application of predictive models. Despite these limitations, our study lays a methodological foundation for future sepsis prediction models after ICH using EMR. To further enhance predictive performance, subsequent studies should integrate static baseline parameters and dynamic physiological trajectories extracted from EMR systems. Embedding a risk calculator within ICU interfaces may be useful for facilitating real-time patient stratification, enabling timely interventions to improve clinical outcomes. Additionally, further randomized controlled trials are warranted to assess whether timely, personalized therapeutic interventions guided by the prediction model can enhance patient outcomes.

### Conclusions

We constructed a web-based risk calculator with 8 features based on the CatBoost model to assist clinicians in identifying people at high risk for sepsis in patients with ICH who are critically ill.

## Appendix

Multimedia Appendix 1 Supplementary figures and tables.

## Abbreviations

AUC: area under the receiver operating characteristic curve
CatBoost: categorical boosting
EMR: electronic medical record
GCS: Glasgow Coma Scale
ICD-9: International Classification of Diseases, Ninth Revision
ICD-10: International Statistical Classification of Diseases, Tenth Revision
ICH: intracerebral hemorrhage
ICU: intensive care unit
LightGBM: light gradient boosting machine
MIMIC-IV: Medical Information Mart for Intensive Care IV
ML: machine learning
MV: mechanical ventilation
RF: random forest
SAPSII: Simplified Acute Physiology Score II
SHAP: Shapley Additive Explanations
SOFA: Sequential Organ Failure Assessment
SpO_2_: percutaneous arterial oxygen saturation
WBC: white blood cell

## Data Availability

The data analyzed and the codes used during this study are available from the corresponding author on reasonable request.
