# Supplementary material for: Identification and Validation of an Explainable Prediction Model of Sepsis in Patients With Intracerebral Hemorrhage: Multicenter Retrospective Study
Source: J Med Internet Res. 2025 Apr 28;27:e71413. doi: 10.2196/71413 (PMC12070006; doi:10.2196/71413)
Supplement: Multimedia Appendix 1 [file jmir_v27i1e71413_app1.docx]

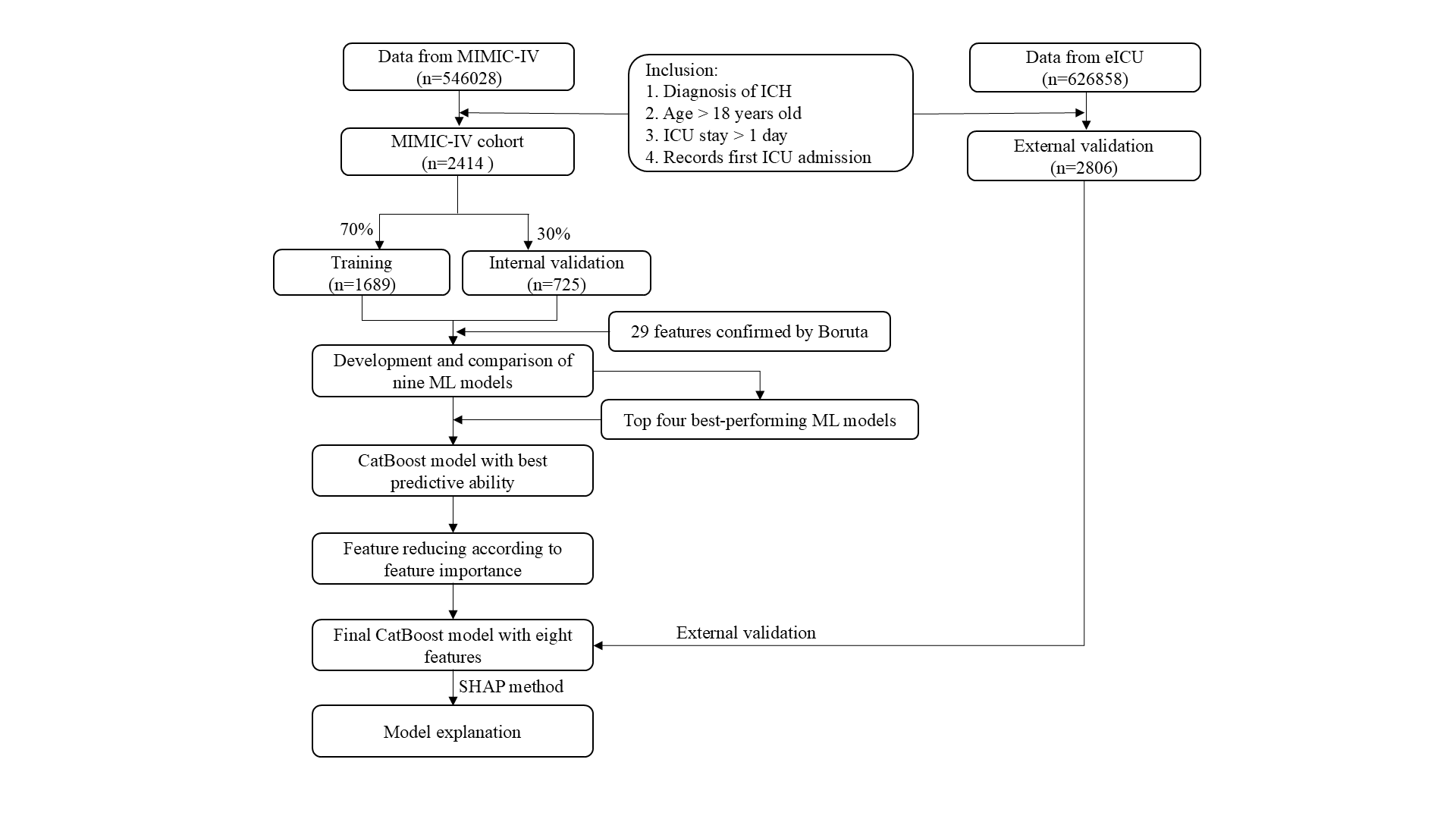


Supplementary Fig. S1 Flow chart of the study design


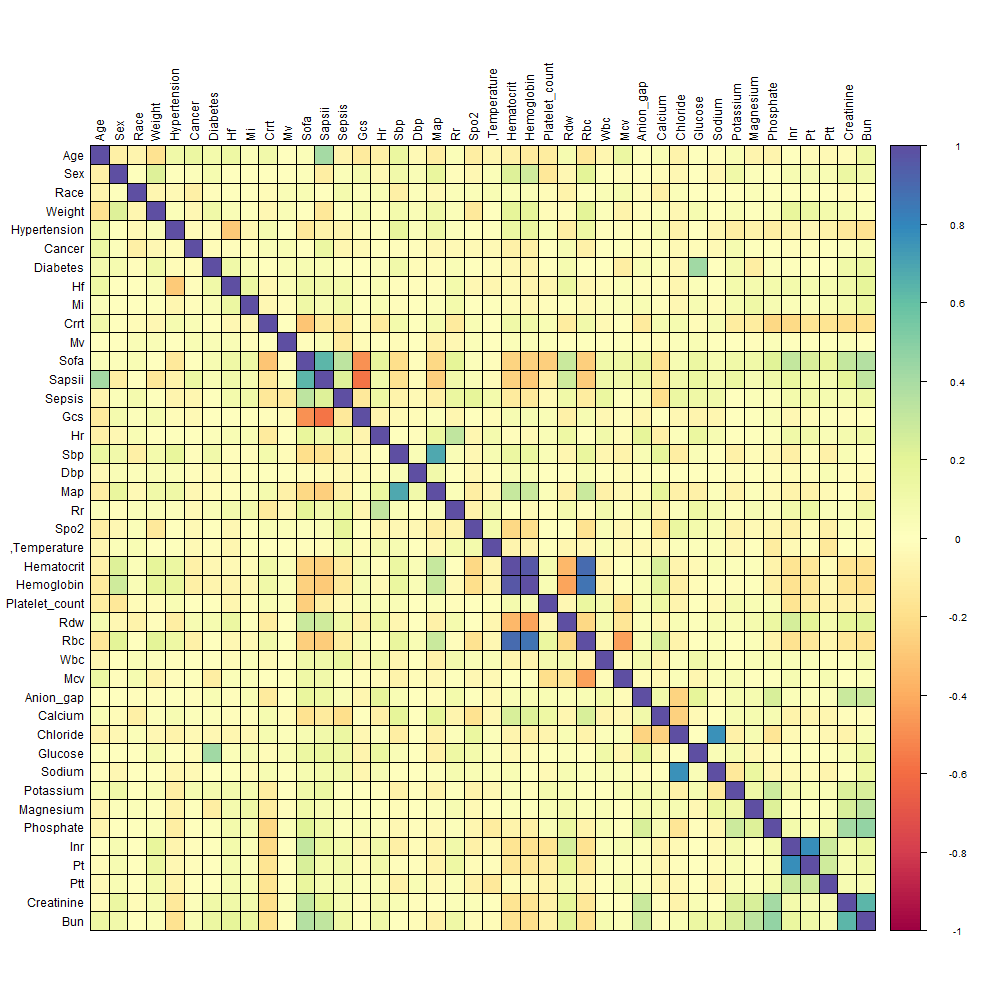


Supplementary Fig. S2 Heat map of Spearman’s correlation analyses among features


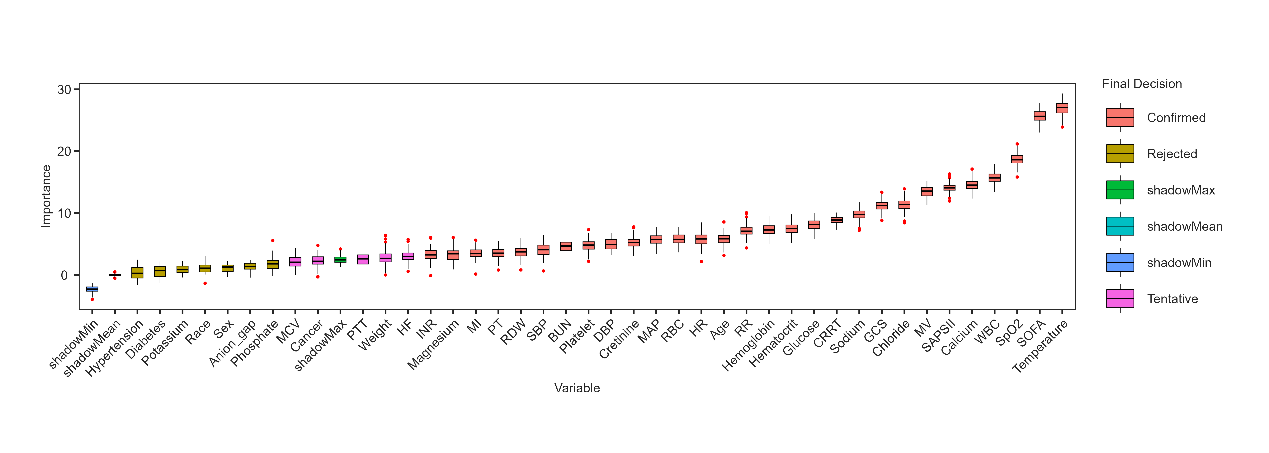


Supplementary Fig. S3 Boruta feature filtering each variable importance box plot


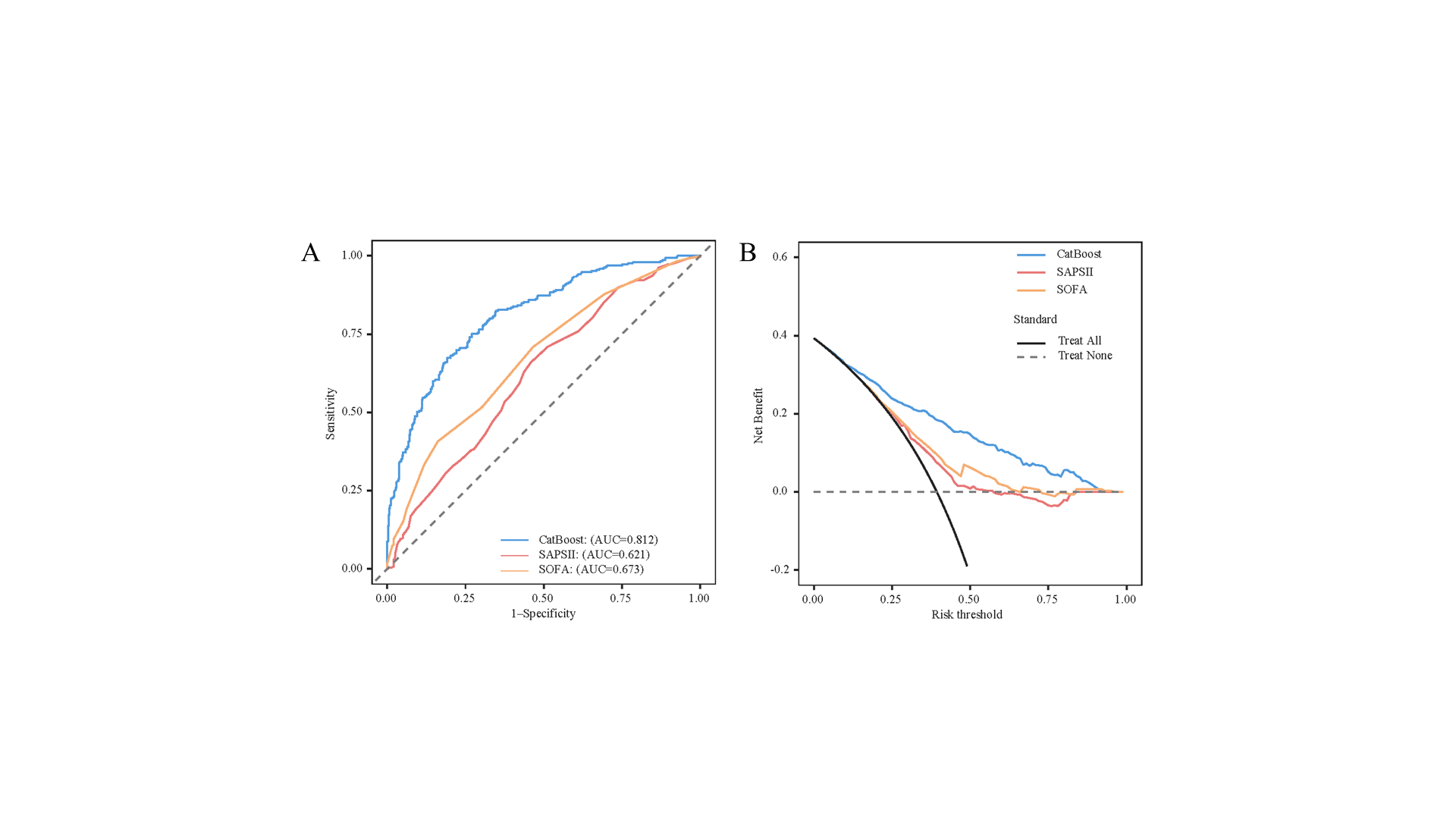


Supplementary Fig. S4 Comparison between final RF model and single features

(A-B) ROC curves (A) and DCA curves (B) of some single features and the final CatBoost model with 8 features.

| Supplementary Table S1 Comparison of demographic and clinical characteristics among the training, internal validation, and external validation sets | | | |
| --- | --- | --- | --- |
| Variable | Training set (n = 1689) | Internal validation set (n = 725) | External validation set (n = 2806) |
| **Demographic** |  |  |  |
| Age, y | 71.0 (59.0 - 81.0) | 69.0 (57.0 - 80.0) | 69 (18.0 - 90) |
| Weight, kg | 76.9 (64.3 - 90.0) | 76.3 (64.5 - 91.8) | 77.2 (25.8 - 200.5) |
| Male, n (%) | 915 (54.2%) | 384 (53.0%) | 1581 (56.3%) |
| Race, n (%) |  |  |  |
| Black | 149 (8.8%) | 67 (9.2%) | 379 (13.5%) |
| White | 990 (58.6%) | 398 (54.9%) | 2193 (78.2%) |
| Other | 550 (32.6%) | 260 (35.9%) | 234 (8.3%) |
| **Clinical severity** |  |  |  |
| SOFA | 3 (2 - 5) | 3 (2 - 5) | 3 (0 - 22) |
| SAPSII | 32 (26 - 40) | 32 (25 - 40) | 30 (0 - 107) |
| GCS | 14 (11 - 15) | 14 (11 - 15) | 14 (3 - 15) |
| **Vital signs** |  |  |  |
| HR, beats/min | 80.0 (70.8 - 89.4) | 78.5 (70.2 - 88.6) | 79.2 (39.3 - 146.7) |
| DBP, mmHg | 70.4 (62.8 - 78.7) | 70.1 (62.6 - 78.3) | 68.5 (35.7 – 129.0) |
| SBP, mmHg | 129.6 (119.6 - 138.8) | 129.6 (118.8 - 138.5) | 132.8 (81.2 - 197.6) |
| MAP, mmHg | 85.8 (77.8 - 93.2) | 85.0 (77.9 - 92.4) | 85.9 (55.0 - 138.8) |
| RR, breath/min | 18.3 (16.5 - 20.5) | 18.4 (16.6 - 20.6) | 18.0 (9.5 - 40.7) |
| SpO2, % | 97.1 (95.8 - 98.6) | 97.2 (95.9 - 98.8) | 97.3 (54.5 - 100.0) |
| Temperature, °F | 37.0 (36.7 - 37.2) | 37.0 (36.8 - 37.283) | 36.9 (36.7 - 37.2) |
| **Laboratory Parameters** | |  |  |
| Hematocrit, % | 36.9 (32.9 - 40.5) | 37.0 (33.3 - 40.2) | 36.5 (10.5 - 61.3) |
| Hemoglobin, g/dL | 12.3 (10.9 - 13.5) | 12.3 (11.0 - 13.5) | 12.2 (3.2 - 21.1) |
| Platelet, 10^9^/L | 205.0 (162.0 - 256.0) | 212.0 (164.7 - 264.0) | 203.6 (2.5 - 771.5) |
| RDW, % | 13.7 (13.1 - 14.7) | 13.8 (13.1 - 14.7) | 14.0 (11.3 - 26.4) |
| RBC, 10^9^/L | 4.1 (3.6 - 4.5) | 4.1 (3.6 - 4.5) | 4.04 (0.9 - 7.3) |
| WBC, 10^9^/L | 10.5 (7.9 - 13.3) | 10.4 (8.2 - 13.4) | 9.9 (0.3 - 290.4) |
| MCV, fl | 91.0 (87.0 - 94.7) | 91.0 (87.0 - 94.3) | 90.2 (60.0 - 123.7) |
| Anion gap, mmol/L | 14.0 (12.0 - 16.0) | 14.0 (12.0 - 16.0) | 10.5 (2.0 – 31.0) |
| Calcium, mg/dL | 8.8 (8.4 - 9.2) | 8.8 (8.4 - 9.1) | 8.6 (5.5 - 11.7) |
| Chloride, mmol/L | 104.0 (101.5 - 107.0) | 104.5 (101.5 - 107.5) | 105.0 (78.0 - 145.3) |
| Glucose, mg/dL | 129.0 (109.0 - 157.0) | 131.0 (110.8 - 158.0) | 127.0 (61.0 – 716.0) |
| Potassium, mmol/L | 3.9 (3.6 - 4.3) | 3.9 (3.7 - 4.2) | 3.9 (2.4 - 6.2) |
| Sodium, mmol/L | 140.0 (137.3 - 142.0) | 140.0 (137.5 - 142.3) | 139.0 (116.7 - 172.0) |
| Magnesium, mg/dL | 2.0 (1.8 - 2.1) | 2.0 (1.8 - 2.1) | 2.0 (0.8 - 3.8) |
| Phosphate, mg/dL | 3.2 (2.7 - 3.7) | 3.2 (2.7 - 3.8) | 3.0 (0.5 - 10.5) |
| INRPT | 1.2 (1.1 - 1.3) | 1.2 (1.1 - 1.3) | 1.1 (0.8 - 4.5) |
| PT, sec | 12.6 (11.7 - 13.9) | 12.6 (11.8 - 13.9) | 13.4 (9.4 - 47.5) |
| PTT, sec | 28.1 (25.7 - 31.2) | 28.2 (25.6 - 31.2) | 28.7 (17.8 - 154.3) |
| Creatinine, mg/dL | 0.9 (0.7 - 1.1) | 0.9 (0.7 - 1.1) | 0.9 (0.2 - 16.9) |
| BUN, mg/dL | 16.0 (12.0 - 22.0) | 16.0 (12.0 - 22.0) | 16.0 (2.0 - 166.3) |
| **Comorbidities** |  |  |  |
| HTN, n (%) | 1074 (63.6%) | 439 (60.6%) | 1622 (57.8%) |
| Cancer, n (%) | 267 (15.8%) | 97 (13.4%) | 2457 (87.6%) |
| Diabetes, n (%) | 422 (25.0%) | 178 (24.6%) | 219 (7.8%) |
| HF n (%) | 205 (12.1%) | 92 (12.7%) | 81 (2.9%) |
| MI, n (%) | 62 (3.7%) | 25 (3.4%) | 77 (2.7%) |
| **Interventions** |  |  |  |
| CRRT, n (%) | 1,661 (98.3%) | 717 (98.9%) | 65 (2.3%) |
| MV, n (%) | 863 (51.1%) | 359 (49.5%) | 846 (30.2%) |

| Supplementary Table S2. Performance of the ML models for sepsis prediction | | | | | | | |
| --- | --- | --- | --- | --- | --- | --- | --- |
| Models | AUC | Sensitivity | Specificity | Accuracy | FPR | FNR | F1 score |
| AdaBoost | 0.655 | 0.639 | 0.671 | 0.658 | 0.330 | 0.361 | 0.595 |
| CatBoost | 0.795 | 0.632 | 0.784 | 0.724 | 0.216 | 0.368 | 0.643 |
| DT | 0.714 | 0.516 | 0.555 | 0.650 | 0.268 | 0.484 | 0.535 |
| KNN | 0.611 | 0.404 | 0.739 | 0.607 | 0.261 | 0.597 | 0.447 |
| LightGBM | 0.796 | 0.649 | 0.775 | 0.726 | 0.225 | 0.351 | 0.650 |
| LR | 0.736 | 0.477 | 0.823 | 0.687 | 0.177 | 0.523 | 0.545 |
| RF | 0.780 | 0.597 | 0.814 | 0.728 | 0.186 | 0.404 | 0.633 |
| SVM | 0.688 | 0.246 | 0.900 | 0.643 | 0.100 | 0.754 | 0.351 |
| XGBoost | 0.771 | 0.614 | 0.768 | 0.707 | 0.232 | 0.386 | 0.623 |
| The indexes represented the performance of ML models in the internal validation set. | | | | | | | |

| Supplementary Table S3. Performance of the CatBoost model with varied numbers of features for sepsis prediction | | | | | | | |
| --- | --- | --- | --- | --- | --- | --- | --- |
| Feature numbers | AUC | Sensitivity | Specificity | Accuracy | FPR | FNR | F1 score |
| 4 | 0.755 | 0.618 | 0.745 | 0.695 | 0.255 | 0.382 | 0.614 |
| 6 | 0.775 | 0.604 | 0.770 | 0.705 | 0.230 | 0.396 | 0.616 |
| 8 | 0.812 | 0.653 | 0.820 | 0.754 | 0.18 | 0.347 | 0.676 |
| 10 | 0.811 | 0.653 | 0.807 | 0.746 | 0.193 | 0.347 | 0.669 |
| 12 | 0.808 | 0.663 | 0.800 | 0.746 | 0.200 | 0.337 | 0.673 |
| 14 | 0.809 | 0.660 | 0.784 | 0.735 | 0.216 | 0.34 | 0.662 |
| 16 | 0.812 | 0.639 | 0.784 | 0.727 | 0.216 | 0.361 | 0.648 |
| 18 | 0.812 | 0.649 | 0.805 | 0.743 | 0.195 | 0.351 | 0.665 |
| 20 | 0.799 | 0.646 | 0.802 | 0.741 | 0.198 | 0.354 | 0.662 |
| 22 | 0.809 | 0.667 | 0.814 | 0.756 | 0.186 | 0.333 | 0.682 |
| 24 | 0.805 | 0.653 | 0.814 | 0.750 | 0.186 | 0.347 | 0.673 |
| 26 | 0.789 | 0.611 | 0.770 | 0.708 | 0.230 | 0.389 | 0.621 |
| 28 | 0.804 | 0.649 | 0.807 | 0.745 | 0.193 | 0.351 | 0.667 |
| The indexes represented the performance of the CatBoost model with varied numbers of features in the internal validation set. | | | | | | | |
